# The utility of the new generation of humanized mice to study HIV-1 infection: transmission, prevention, pathogenesis, and treatment

**DOI:** 10.1186/1742-4690-8-65

**Published:** 2011-08-11

**Authors:** Bradford K Berges, Mark R Rowan

**Affiliations:** 1 Department of Microbiology and Molecular Biology, Brigham Young University, Provo, UT 84602, USA

## Abstract

Substantial improvements have been made in recent years in the ability to engraft human cells and tissues into immunodeficient mice. The use of human hematopoietic stem cells (HSCs) leads to multi-lineage human hematopoiesis accompanied by production of a variety of human immune cell types. Population of murine primary and secondary lymphoid organs with human cells occurs, and long-term engraftment has been achieved. Engrafted cells are capable of producing human innate and adaptive immune responses, making these models the most physiologically relevant humanized animal models to date. New models have been successfully infected by a variety of strains of Human Immunodeficiency Virus Type 1 (HIV-1), accompanied by virus replication in lymphoid and non-lymphoid organs, including the gut-associated lymphoid tissue, the male and female reproductive tracts, and the brain. Multiple forms of virus-induced pathogenesis are present, and human T cell and antibody responses to HIV-1 are detected. These humanized mice are susceptible to a high rate of rectal and vaginal transmission of HIV-1 across an intact epithelium, indicating the potential to study vaccines and microbicides. Antiviral drugs, siRNAs, and hematopoietic stem cell gene therapy strategies have all been shown to be effective at reducing viral load and preventing or reversing helper T cell loss in humanized mice, indicating that they will serve as an important preclinical model to study new therapeutic modalities. HIV-1 has also been shown to evolve in response to selective pressures in humanized mice, thus showing that the model will be useful to study and/or predict viral evolution in response to drug or immune pressures. The purpose of this review is to summarize the findings reported to date on all new humanized mouse models (those transplanted with human HSCs) in regards to HIV-1 sexual transmission, pathogenesis, anti-HIV-1 immune responses, viral evolution, pre- and post-exposure prophylaxis, and gene therapeutic strategies.

## Review

### Introduction to humanized mice

Humanized mice have allowed for extensive study of the development and function of the human immune system. Soon after their inception, the SCID-hu *thy/liv *[1] and SCID-hu-PBL [2] models (pioneered by McCune and Mosier, respectively) were shown to support infection of pathogens that replicate in human immune cells. In particular, Human Immunodeficiency Virus Type 1 (HIV-1) infection has been studied in great detail for over two decades in humanized mice, largely due to the expense involved with the use of non-human primates and key differences between HIV-1 infection in humans and chimpanzees. HIV-1 infection of humanized mice has yielded valuable data ranging from the fields of *in vivo* pathogenesis to drug efficacy and passive immunity. However, there are caveats in the original humanized mouse models: only a limited hematopoietic repertoire was engrafted which varied by model, HIV-1 infections were often short-term, and no primary adaptive immune response was mounted against HIV-1 [3-6]. Thus, these models provided data on acute HIV-1 infection of limited cell types and virtually unopposed by a host immune response.

Recent advances in the production of profoundly immunodeficient mouse strains have resulted in improved human cell engraftment relative to the original SCID-hu *thy/liv *and SCID-hu-PBL models [1,2]. The C.B-17 SCID mouse (*Prkdc *mutation) used in the original humanized mouse models can spontaneously generate murine T and B cells as animals age (leakiness) and has high levels of natural killer (NK) cell activity, both of which prevent efficient/prolonged xenoengraftment [7]. Improvements in the available immunodeficient mouse strains and the use of human CD34^+ ^hematopoietic stem cells (HSCs) have resulted in improved, long-term engraftment of a variety of human hematopoietic cell types as well as the ability to generate primary human immune responses [8].

Mice deficient in the recombinase activating genes 1 and 2 (Rag1 and Rag2, respectively) do not exhibit leaky production of T and B lymphocytes. The immune phenotypes in Rag1^-/- ^and Rag2^-/- ^strains are similar [9,10]. However, Rag-deficient animals produce normal levels of NK cells, and thus additional mutations are required in order to produce animals better suited for xenoengraftment studies. The non-obese diabetic (NOD) SCID mouse is commonly used because the *Prkdc *mutation prevents formation of mature T and B lymphocytes while the NOD mutation results in a reduction of NK cell activity [11]. However, it should be noted that a major disadvantage of the NOD strain is a high incidence of spontaneous thymic lymphomas which results in a shortened lifespan [12]. The common gamma chain receptor (γc, also referred to as the IL-2 receptor gamma chain) is a component of the IL-2, IL-4, IL-7, IL-9, IL-15, and IL-21 receptors and is the gene involved in X-linked SCID [13]. The addition of the γc mutation to the Rag1, Rag2, and NOD/SCID backgrounds further blocks T and B cell development due to a lack of IL-2 signaling and also prevents maturation and expansion of NK cells via a lack of IL-15 signaling [14,15]. Since *Prkdc*^-/-^ (SCID) animals can experience leaky production of T and B cells, the γc mutation is useful as a secondary means to block maturation of these cells. Nearly all current humanized mouse models use γc^-/- ^animals, with the exception being NOD/SCID animals which are used for either HSC engraftment or to produce the BLT model (see below). It should be noted that there are two different γc mutations commonly in use for production of humanized mice. One is a null mutation [16], and the other is a truncation of the cytoplasmic signaling domain [14]. It is currently unclear if there are functional differences between these two mutations, since both are expected to block signaling. In combination with the NOD/SCID mutation, these mouse strains are commonly referred to as NOG (truncation) and NSG (knockout) mice.

Combinations of the above mutations thus far analyzed for human hematopoietic stem cell engraftment have included such strains as Rag2^-/-^γc^-/-^, NOD/SCID, NOD/SCIDγc^-/-^, Rag1^-/-^γc^-/-^, NOD/Rag1^-/-^γc^-/-^, and NOD/SCIDβ2 m^-/- ^mice [17-23]. When engrafted with hematopoietic stem cells, these immunocompromised strains have shown the most effective xenoengraftment observed to date, in terms of the spectrum of human cells produced, the penetration of human cells into various organs, the duration of engraftment, and the ability to generate primary human adaptive immune responses [8]. All of the major HIV-1 target cells, including human CD4^+ ^T cells, monocytes, macrophages, and dendritic cells are readily detected in these humanized mice. A detailed review of how to generate humanized Rag2^-/-^γc^-/- ^mice is available [24], and methods to prepare humanized NOD/SCIDγc^-/- ^mice and humanized Rag1^-/-^γc^-/- ^mice are very similar. It should be noted that the methods used to prepare human HSCs and to inject animals with grafts are relatively straightforward and in many cases require a simple, intrahepatic or intravenous injection. The BLT model (bone marrow, liver, thymus) engrafts NOD/SCID or NOD/SCIDγc^-/- ^mice with a combination of human fetal liver and thymic tissue (the SCID-hu *thy/liv* model) followed by a CD34^+ ^cell graft. As a result of the relatively simple techniques needed to generate humanized mice, many new laboratories are adopting these models.

### Viral infections in humanized mice

Improved and prolonged human cell engraftment has generated renewed interest in the study of human pathogens of the immune system in the hopes that previous problems have been solved (see first paragraph). To date, the newer humanized mouse models have been examined for infection by a variety of human viruses including the retroviruses HIV-1 (subject of this review) and HTLV-1 [25]; the herpesviruses EBV [17,26-30], KSHV [31,32], hCMV [33], and HSV-2 [34]; Dengue virus [35,36]; and recombinant adenovirus [37]. An adaptation using human liver transplants has also been described [38,39] which can be infected with HBV and HCV [40,41]. A summary of all viral pathogens and other immunogens studied thus far in the new generation of humanized mice is provided in Table 1.

**Table 1:** Viruses and other immunogens studied in the new generation of humanized mice

Infecting Agent	Humanized mouse model	References
**Viruses:**		

HIV-1	Rag2-/-γc-/-	[44,46,48-51,55-61,63-65]

HIV-1	NOD/SCIDγc-/- (hNOG)	[43,66,67,72,73]

HIV-1	NOD/SCIDγc-/- (hNSG)	[68-71,74-76]

HIV-1	NOD/SCID BLT	[42,45,77-79]

HIV-1	NOD/SCIDγc-/- (hNSG) BLT	[42,81]

HIV-1	Rag1-/-γc-/-	[23]

HTLV	NOD/SCID	[25]

EBV	Rag2-/-γc-/-	[17,122]

EBV	NOD/SCIDγc-/- (hNOG)	[27,28]

EBV	NOD/SCIDγc-/- (hNSG)	[29]

EBV	NOD/SCID BLT	[30]

EBV	NOD/SCID	[26]

KSHV	NOD/SCID	[31,32]

Dengue Virus	Rag2-/-γc-/-	[35]

Dengue Virus	NOD/SCID	[36]

HSV-2	Rag2-/-γc-/-	[34]

rAd-HCV	NOD/SCIDγc-/- (hNSG)	[37]

hCMV	NOD/SCIDγc-/- (hNSG)	[33]

HCV	Rag2-/-γc-/-Fah-/-	[40]

HBV	Rag2-/-γc-/-Fah-/-	[40]

HBV	Rag2-/-γc-/-uPa-/-	[123]

**Bacteria:**		

Salmonella typhi	Rag2-/-γc-/-	[124]

**Non-infectious agents:**		

Tetanus toxoid	Rag2-/-γc-/-	[17,97]

Haemophilus influenza B conjugate vaccine	Rag2-/-γc-/-	[46]

Hepatitis B surface antigen vaccine	Rag2-/-γc-/-	[97]

Toxic shock syndrome toxin 1	NOD/SCID BLT	[30]

2,4-dinitrophenyl hapten-keyhole limpet hemocyanin	NOD/SCID BLT	[47]

One of the most exciting developments for the new generation of humanized mice is the generation of primary human adaptive immune responses following infection or immunization with a variety of pathogens. T cell responses have been demonstrated against HIV-1, EBV, toxic shock syndrome toxin 1, and a recombinant adenoviral vector expressing HCV proteins [17,28-30,37,42], while B cell responses have been documented against HIV-1, Dengue virus, tetanus toxoid, KSHV, HSV-2, and the haemophilus influenzae B conjugate vaccine [17,32,34,35,42-46]. Antigen-specific antibody class-switching and detection of neutralizing antibody responses following Dengue virus infection illustrates the potency of the human adaptive immune response generated in these new and improved models [35,47].

### Humanized mouse models and HIV-1 strains used to infect them

To date, six different mouse strains humanized with human HSCs have been analyzed for HIV-1 infection, including Balb/c Rag2^-/-^γc^-/- ^mice (RAG-hu) humanized with purified CD34^+ ^HSCs derived from umbilical cord blood [44,48-53], from fetal liver [48,49,54-64] or from a non-specified source [65]; NOD/SCID γc^-/- ^(hNOG or hNSG) mice humanized with cord blood derived CD34^+ ^cells [43,66-75]; hNOG or hNSG mice humanized with fetal liver derived CD34^+ ^cells [76]; NOD/SCID BLT mice [42,45,77-80], hNSG BLT mice [42,81], and Balb/c Rag1^-/-^γc^-/- ^mice humanized with purified CD34^+ ^HSCs from human fetal liver [23]. One report has shown successful engraftment and HIV-1 infection in C57BL/10 Rag2^-/-^γc^-/- ^mice engrafted with non-purified human fetal liver cells [64], although another report has shown an inability to achieve usable engraftment in the related C57BL/6 Rag2^-/-^γc^-/- ^strain [34]. Two reports examining RNA interference strategies against HIV-1 have used *ex vivo *infections of humanized mouse-derived cells [60,79].

A large variety of HIV-1 isolates have been examined for infection in the new humanized mouse strains, although most work has been performed with molecular clones of the virus. The following CCR5 tropic strains have been reported: JR-CSF [42,43,48,49,52,53,59,64-68,72,77,78], BaL-1 [23,55,57,62,69,70,74], YU-2 [44,48,51], ADA [46,71,75,76], NFN-SX(SL9) [58,79], 1157 [46], a *vif*-deficient strain of JR-CSF [73], and the NL4-3 strain but with the *env *gene replaced with the BaL-1 *env *sequence [70]. The following CXCR4 tropic strains have been studied: NL4-3 [23,44,48,49,54,55,57,67,72,79], LAI [45], MNp [67], a variant of NL4-3 with GFP inserted (NLENG1-IRES) [57], and LAI with a V38E mutation in gp41 [80]. The following dual-tropic strains have additionally been analyzed for infection: HIV-R3A [49,61,63] and 89.6 [48]. One study has used a chimeric Simian Immunodeficiency Virus encoding the envelope gene from the dual-tropic HIV-1 strain 89.6 (SHIV-C2/1) [43]. Finally, primary isolates (not molecular clones) from subtype B (strain UG 209A) and subtype C (strain 1157) have also been examined in RAG-hu mice, although UG 209A was only tested in mice humanized with human PBLs. It is clear from the results that HSC-humanized mice are susceptible to a broad variety of HIV-1 strains. Further work should involve diverse primary isolates of HIV-1, especially when the efficacy of vaccination is under examination. Since virus stocks derived from molecular clones are identical or nearly identical in genomic sequence, the ability to block viral transmission is predicted to be much simpler as compared to the natural scenario in humans where exposure is to multiple genetically distinct isolates [82]. Additionally, little work has been done using mutant strains of HIV-1 to discover the contribution of various genes to pathogenesis, although the few reports to do so are summarized herein.

We are not aware of any studies that have examined the minimum level of human cell engraftment required to achieve consistent HIV-1 infection, although in our hands we have found that an animal with as low as 5% peripheral blood engraftment (5% hCD45^+^, 95% mCD45^+^) can be infected by intraperitoneal injection [57]. A minimal dose of virus required to infect humanized mice has also not been established, although unsuccessful infections using direct injection routes with low doses of HIV-1 (100-500 TCID_50_) have been reported [46,58]. 1 ng of p24 was sufficient to infect all RAG-hu animals in two studies from the same group [49,61]. For nearly all studies the goal has been to achieve successful viral replication *in vivo* and the impact (if any) of the infectious dose has not been explored, despite a large range of doses studied ranging from 10^2 ^TCID_50 _[46] or 1 ng p24 [49,61] to 2 × 10^6 ^TCID_50 _or 400 ng p24. Mucosal transmission has been achieved with a dose as low as 156 TCID_50 _in RAG-hu mice or 170 ng p24 in BLT mice. When molecular clones of the virus are used for infections, the impact of the infectious dose may not be as critical to many experiments as compared to a highly diverse population. Since engraftment levels vary from one animal to another and infection routes differ by study, it is not anticipated that a uniform minimal dose will apply to all animals or to all engraftment models.

### Routes of viral infection

Various routes of viral exposure have been tested in humanized mice. Direct routes such as intravenous [43,49,59,61,64,65,67,78], intraperitoneal [42,44,50,51,57,58,64,66,69,71,72] and intrasplenic [64] injections have been used extensively and result in a very high efficiency of infection. In addition, mucosal engraftment of human HIV-1 target cells has been documented; also, mucosal transmission of CCR5 tropic viruses across an intact epithelium has been found in both the BLT and RAG-hu models. Abrasions have been used for rectal transmission in BLT mice [45,78], possibly to mimic rectal intercourse, but abrasions are not required for rectal transmission in RAG-hu mice [55]. Abrasions are not necessary for vaginal transmission in BLT mice [77], RAG-hu mice [55], or humanized Rag1^-/-^γc^-/- ^mice [23]. We have previously shown successful mucosal transmission in RAG-hu mice for both CCR5 tropic and CXCR4 tropic strains introduced both vaginally and rectally, although a lower rate of infection was observed with CXCR4 strains which are not effectively transmitted sexually in humans [55].

It should be noted that poor intestinal engraftment and only rare HIV-1 rectal transmission in RAG-hu mice were reported by Hofer *et al*. [48]. Additional reports have shown mucosal engraftment in RAG-hu mice [34,59] and HSC-engrafted hNSG mice [74]. These findings indicate that simple HSC engraftment is sufficient to achieve mucosal engraftment as compared to the thymic implants required for the BLT model. In addition, HIV-1 nucleic acids were detected in the rectum, small intestine, and uterus of infected hNOG mice [67], although it is not clear if this is due to the presence of blood cells in non-perfused mice. Other reports have shown successful vaginal HIV-1 transmission in RAG-hu mice without examining mucosal engraftment [62,83]. Finally, in our recent work with humanized Rag1^-/-^γc^-/- ^mice we have also detected successful vaginal transmission of HIV-1 [23]. These data indicate that mucosal engraftment and HIV-1 transmission are possible in a wide variety of mouse strains, and that simple HSC implants are sufficient (as compared to thymic implants plus HSC implants in BLT mice).

The reasons for the discrepancy in detection of human cell engraftment in the mucosa of humanized mice and/or differences in HIV-1 transmission rates are still unclear. However, several variables between the various experiments could suggest possible explanations for differing results. These include the source of cells (fetal liver vs. cord blood), the strains of mice used, cytokine-mediated expansion of HSCs, analysis of vaginal vs. rectal engraftment/transmission, and the use of antibiotics for mouse maintenance. First, some papers that have analyzed mucosal engraftment and/or HIV-1 transmission have used cord blood [34,67,74] and others have used fetal livers [23,45,55,59,62,77,78,83] as sources of HSCs; two papers have examined both types of cells [48,59]. Since fetal liver cells are more primitive than cord blood cells (they are taken from pre-term samples and cord blood is taken from full-term samples), it is possible that they have a greater capacity for repopulation, differentiation, and organ penetration. It is clear that the use of fetal liver cells results in mucosal engraftment [45,55,77] and mucosal HIV-1 transmission [23,55,62,77,78,83]. The data for cord blood cells are not as clear. Holt *et al*. used cord blood cells in hNOG mice and frequently detected human cell engraftment in the large and small intestines (including CD4^+ ^T cells) but did not attempt mucosal transmission [74]. It is important to note that Holt *et al*. used cytokines to mediate HSC expansion in preparation for nucleofection. It has been suggested that cytokine-mediated expansion of HSCs may explain the differing levels of mucosal engraftment [59]. The putative mechanism of this hypothesis is that the use of cytokines modifies the properties of HSCs, thereby giving them an increased potential for differentiation and penetration into mucosal tissues. The report by Hofer et al. analyzed rectal and intestinal tissues and used either fetal liver or cord blood cells as HSCs, but detected little engraftment and only rarely achieved rectal transmission [48]. No cytokines were used to expand either type of cell. The use of cytokines to expand HSCs is thus a critical difference between the methods used by Hofer *et al*. and others. Choudhary *et al*. used fetal liver cells and also cultured HSCs in the presence of cytokines and detected significant intestinal engraftment in 2 of 6 mice [59]. Papers from the Akkina group have all used fetal liver cells and cytokine expansion [23,55,62,83]. Kwant-Mitchell *et al*. used cord blood cells that were not expanded with cytokines and only detected mucosal engraftment after vaccination with attenuated HSV-2 [34]. A recent study indicates that engraftment of the peripheral blood, lymph nodes, spleen, and bone marrow of RAG-hu mice is significantly higher after culturing HSCs in the presence of cytokines versus immediate transplantation of uncultured cells [84]. Serum IgG levels are also significantly higher, although IgM levels were not [84]. This study supports the hypothesis that culturing HSCs in the presence of cytokines prior to engraftment leads to significantly better engraftment as assessed by multiple endpoints, but no analysis was made of mucosal engraftment. Thus, it appears that the use of cytokines to expand HSCs may be critical to achieving mucosal engraftment, even more so than the use of fetal liver cells.

The mouse strain used does not appear to make a difference in the ability to achieve mucosal engraftment or HIV-1 mucosal transmission with the data currently available. Mucosal engraftment has been detected in NOD/SCID BLT mice [45,77], Rag2^-/-^γc^-/- ^mice [34,55,59], and hNOG mice [74] while HIV-1 mucosal transmission has been detected in NOD/SCID BLT mice [45,77,78], Rag2^-/-^γc^-/- ^mice [55,62,83], and Rag1^-/-^γc^-/- ^mice [23]. Interpretations of mucosal engraftment and susceptibility to infection should also recognize that vaginal and rectal/intestinal engraftment cannot be directly compared. Hofer *et al*. focused their study on rectal engraftment in RAG-hu mice and did not examine vaginal engraftment and transmission. Of the studies to emerge from the Akkina lab, only one examined rectal engraftment and transmission [55] while most studies have focused on vaginal transmission [23,55,62,83] with a single report that evaluated vaginal engraftment [55]. While we are confident in our ability to achieve rectal engraftment and transmission, the sample size of our vaginal challenge experiments is much higher and vaginal engraftment and transmission were not examined by Hofer *et al*.

A final point to consider is the status of the normal microbial flora of the humanized mouse rectum and intestinal tract. Humanized mice are sometimes housed on a regimen of antibiotics in their drinking water in order to prevent bacterial infections that are common in highly immunodeficient mice. It is possible that various mouse colonies are maintained with different antibiotics and/or concentrations of antibiotics, or no antibiotics at all, thus leading to differences in the bacteria present in the gut. The flora present (or lacking) in various colonies may influence the ability and/or tendency of human immune cells to traffic to mucosal sites.

### HIV-1 viremia

Viremia in HSC-humanized mice is usually detectable by 1 week post-infection, which often represents the first time point analyzed. The most sensitive means to detective viremia is quantitative PCR (Q-PCR), since comparable studies using p24 ELISA have shown an inability to detect viremia at various time points post-infection [46,50,58] whereas Q-PCR shows consistent detection [44,56]. The mean peak viremia for CXCR4 virus is approaching 10^7 ^viral genomes per ml of plasma while the mean peak viremia for CCR5 virus is around 10^6 ^viral genomes per ml of plasma [43-45,50,53,55,56,67,72,75,77,78]. Peak viremia typically occurs in the range of 1-2 months post-infection [23,44,45,53,55,56,67,72,74]; however, CD4^+ ^T cell loss in the blood is more severe with CXCR4 virus and the decrease in target cells is accompanied by a decrease in viremia [23,44,56]. CCR5 tropic virus maintains a higher level of viremia for sustained periods which correlates with decreased levels of target cell loss over time. The peak level of viremia, both in terms of kinetics and levels, is similar for all routes of infection and no significant differences are apparent by humanized mouse model. The impact of viral dose on subsequent viral loads is still unclear due to the presence of multiple variables between experiments, including methods used to titer virus stocks. However, animals infected by mucosal transmission tend to show sporadic detection of viremia for the first weeks of infection [23,45,77,78]. We have shown that HIV-1 infection in RAG-hu mice can be sustained for over a year post-infection with either CCR5 tropic or CXCR4 tropic virus. We detected viremia for up to 63 weeks and HIV-1 RNA by in situ hybridization for up to 67 weeks post-infection [56]. Some groups have reported a drop to undetectable levels of viral load for multiple time points, but this is a rare finding occurring in few mice [44,72]. Most reports consistently detect all infected animals to be positive [23,55-57,67,78,85]. Thus, not only is human cell engraftment a life-long condition in HSC-humanized mice, but productive HIV-1 infection is also maintained for the same period. Since many HIV-1-associated diseases take time to develop, it is important that these models sustain long-term infections.

### Sites of virus replication

The distribution of HIV-1 replication in humanized mice has been analyzed by many independent groups using immunohistochemical and *in situ *hybridization techniques to detect HIV-1 proteins and genomes/gene expression, respectively. The most prominent organs that feature HIV-1 replication in humans are also highly positive for HIV-1 replication in humanized mice, namely the spleen [42-46,49,52,55,57,72,77,78], lymph nodes [42-46,49,55,56,71,72,77], and thymus [44,49,55,57]. The thymic organoid graft of BLT mice is also highly positive for viral replication [45,77,78]. In addition, the level of human cell engraftment in other organs is also sufficient to sustain detectable HIV-1 replication, including the bone marrow [56,67,72], lungs [43,45,67,77], small intestines [45,55,67], large intestines [45,78], male reproductive tract [45], and female reproductive tract [45,67,77]. One recent study has shown that human macrophages can be detected in hNSG brains and that systemic infection with HIV-1 leads to detection of p24^+ ^cells in the hNSG brain [75]. The distribution of engrafted cells and HIV-1 replication in HSC-engrafted humanized mice is impressive and indicates that these models are superior to the previous SCID-hu *thy/liv *and SCID-hu PBL models in terms of penetration of the graft into various tissues, accompanied by the ability of HIV-1 to traffic to and replicate in various sites. As expected, CCR5 tropic strains are largely unable to replicate in the humanized mouse thymus due to the immature, CCR5-negative status of human thymocytes [44,86]. When the identity of p24^+ ^cells has been analyzed, most cells have been shown to be CD4^+ ^T cells although infected CD68^+ ^macrophages have also been detected [44,72].

### CD4^+ ^T cell loss occurs in blood, lymphoid organs, and gut-associated lymphoid tissue

CD4^+ ^T cell loss in the blood occurs in every HSC-engrafted model and with nearly every virus strain analyzed to date. Loss occurs regardless of the route of infection, including mucosal transmission. In one case, CCR5-tropic HIV-1 failed to deplete in the hNOG model through 6 weeks of infection [43], but all other studies using strain JR-CSF have shown successful loss [42,45,49,51,59] unless antiviral strategies were employed. Gorantla *et al*. showed a lack of CD4^+ ^T cell loss with a CCR5 tropic primary isolate (subtype C strain 1157), but it is unclear what time point was analyzed, and if animals were monitored long enough to detect loss with a CCR5-using strain [46]. It is also possible that CD4^+ ^T cell rebound (see below) had occurred at the time point analyzed.

Some reports with mutated virus strains have shown an inability to deplete helper T cells levels. However, the study of mutant viral strains in humanized mice is still underdeveloped when the large body of mutant viruses examined in tissue culture is considered. Our first report in RAG-hu mice showed a lack of CD4^+ ^T cell loss in the blood through 24 weeks of infection with NLENG1-IRES, a reporter strain of HIV-1 that expresses GFP and places the *nef *gene under the control of an IRES element in the background of the CXCR4-tropic strain NL4-3 [87]. In this virus, *nef *gene expression is not under the control of the native viral promoter, and gene function may be attenuated. Two mice were reported [57], but follow-up work with an additional 3 mice gave the same result (unpublished). Viremia was readily detectable in these mice, but the lack of T cell loss may indicate that attenuation of *nef *gene expression in HIV-1 is able to prevent viral pathogenesis and AIDS. Interestingly, no differences in viremia have been noted between animals infected with NL4-3 and NLENG1-IRES, suggesting that viral replication occurs in similar fashion regardless of *nef *gene status, but that patterns of CD4^+ ^T cell loss in the blood may be different.

Another study with a lethal *vif *mutation in the CCR5 tropic JR-CSF background showed an inability to replicate or to cause CD4^+ ^T cell depletion in hNOG mice; this finding was likely due to increased susceptibility of the virus to human APOBEC proteins which are normally targeted for degradation by vif [73]. HIV-1 *vif *is known to promote destruction of human APOBEC3G *in vitro*, but *in vivo* studies were needed to confirm the relevance. hNOG mice were infected with either wild-type virus or a *vif*-deficient strain and hypermutations induced by the cytosine deaminase activity of human APOBEC proteins were detected specifically in cells infected by the *vif*-deficient strain. Together, these reports provide evidence that targeting of specific HIV-1 proteins for mutation or silencing can either block virus replication or virus-induced pathogenesis.

As mentioned above, CD4^+ ^T cell loss in blood is both more rapid and more severe with CXCR4-virus or dual-tropic virus. However, CD4^+ ^T cell rebound has been shown to occur in some studies, with CCR5-virus [23,43,44,53,55,57,58] or CXCR4-virus [23,44,55,57] or dual-tropic virus [49]. Other reports have failed to detect CD4^+ ^T cell rebound, with most using CCR5-virus [46,67,74,77,78] and two using CXCR4-virus [45,67]. The mechanism for CD4^+ ^T cell rebound is currently unclear, but this rebound has been seen in some studies that track infection for at least several months. Immune responses are one possible reason, but murine adaptive immunity is absent, and human anti-HIV-1 adaptive immunity is not thought to be very strong in most HSC-engrafted models (see below). The drop in CD4^+ ^T cells is accompanied by a drop in viremia, and this decrease in total virus levels may explain the ability of helper T cells to rebound. Direct routes of infection (intraperitoneal or intravenous) give similar results for CD4^+ ^T cell loss, while limited data on mucosal transmission indicate that slower and less severe loss occurs [45,55,77].

CD4^+ ^T cell loss in the blood and lymphoid organs do not always correlate with one another. Several groups have examined CD4^+ ^T cell levels in primary and secondary lymphoid organs of HSC-humanized mice in order to determine the extent of CD4^+ ^T cell loss. Zhang *et al*. showed loss of CD4^+ ^T cells by FACS analysis in the lymph nodes and thymus of RAG-hu mice infected with dual-tropic virus by FACS staining, with CCR5 tropic virus unable to deplete CD4^+ ^thymocytes. Severe loss of CD4^+^CD8^+ ^thymocytes was seen with dual-tropic virus [49]. Jiang *et al*. reported that regulatory helper T cells are specifically depleted in spleen and mesenteric lymph nodes relative to other types of helper T cells early during infection of RAG-hu mice by FACS analysis [61]. Our group showed that CD4^+ ^thymocytes are depleted in large areas of the RAG-hu thymus following infection by CXCR4-tropic virus by immuno-staining [57]. Sun *et al*. showed intrarectal HIV-1 transmission which was accompanied by an overall decrease in hematoxylin and eosin-stained cells in the thymic organoid and small and large intestines in mice infected with a CXCR4-tropic strain [45]. They also used FACS staining to show CD4^+ ^T cell loss in the bone marrow, thymic organoid, spleen, peripheral and mesenteric lymph nodes, liver, lung, and small and large intestines with either CXCR4-virus [45] or CCR5-virus [77]. The extensive loss of helper T cells in the gut-associated lymphoid tissue (GALT) is comparable to human AIDS patients and indicates that BLT mice, and possibly other strains of HSC-humanized mice, are a useful model to study HIV-1 pathogenesis associated with GALT infection.

### Mechanisms of HIV-1 pathogenesis

Due to the lack of appropriate *in vivo* models required to study HIV-1-mediated pathogenesis, there are still many unanswered questions about how HIV-1 infection leads to AIDS. Several research groups have begun to analyze the mechanisms of HIV-1-mediated pathogenesis in HSC-humanized mice, and it is clear that at least some mechanisms proposed to take place in humans also occur in HSC-humanized mice [51,61,63,72,75,76,80]. A summary of mechanisms of HIV-1 pathogenesis explored to date in humanized mice is found in Table 2.

**Table 2:** Mechanisms of HIV-1 pathogenesis in the new generation of humanized mice

Mouse Model/HIV-1 strain	Finding	Reference
RAG-hu mice; R5 tropic JR-CSF and dual-tropic R3A	CD4^+^FoxP3^+ ^T regulatory cells are preferentially infected and depleted in spleen and lymph nodes; depletion occurs via apoptosis.	[61]

hNOG mice; R5 tropic JR-CSF or X4 tropic NL4-3	X4 tropic virus depletes both naïve and memory T cells, while R5 tropic virus selectively depletes effector memory T cells (CD45RO^+^CD45RA^-^).	[72]

BLT mice; R5 tropic JR-CSF	R5 tropic virus depletes CD4^+ ^effector memory T cells (CD45RA^-^CD27^-^) in small intestines	[77]

RAG-hu mice; R5 tropic YU-2	R5 tropic virus leads to translocation of LPS to the plasma, resulting in CD8 T cell activation, lower CD4 T cell ratios, and higher viral loads.	[51]

hNOG mice; R5 tropic ADA	Human macrophages, microglia, and dendritic cells are engrafted in the meninges and perivascular spaces in the hNOG brain. p24+ cells can be detected in the brain following intraperitoneal infection. Human immune cells infiltrate regions of viral replication in the brain, and CD8 T cell depletion leads to meningitis and encephalitis.	[75]

hNOG mice;R5 tropic ADA	HIV-1 infection leads to structural changes in brain architecture, leading to loss of neuronal integrity.	[76]

RAG-hu mice; dual-tropic R3A	Plasmacytoid dendritic cells (pDC) are productively infected and activated during early HIV infection, leading to CD4 T cell activation and apoptosis. pDC levels were stable, but function was impaired in the spleen and bone marrow.	[63]

BLT mice; NL4-3 backbone with LAI *env *gene	Virus with a single amino acid substitution in *env *(V38E) has similar viral load to virus with wild-type *env*, but is attenuated for CD4 T cell depletion due to a defect in caspase-dependent bystander apoptosis.	[80]

A hallmark of AIDS is chronic immune activation, and specific infection and depletion of regulatory T cells (T-regs), which normally suppress chronic immune cell activation, could play a critical role in this process. Jiang *et al*. reported that CD4^+^FoxP3^+ ^T-regs develop in RAG-hu mice. These T-regs migrate to all lymphoid organs and are functional in that they can suppress proliferation of other T cells. T-regs are preferentially infected and depleted *in vivo* in RAG-hu mice as compared to other types of human T cells; both the CCR5 tropic JR-CSF and the dual-tropic HIV-R3A exhibited the increased rate of infection. Specific depletion of T-regs was largely due to apoptosis. Depletion of T-regs by administration of denileukin diftitox led to reduced viral replication as measured by viral load and presence of p24^+ ^cells [61].

Depletion of effector memory T cells may lead to exhaustion of the central memory T cell pool, which has been postulated to play a role in progression to AIDS [88]. Nie *et al*. examined the effects of HIV-1 infection on memory T cell populations in hNOG mice [72]. They showed that while CXCR4 tropic HIV-1 is able to deplete both naïve and memory T cells in hNOG mice, CCR5 tropic HIV-1 selectively depletes effector memory T cells. Similar findings with CCR5 tropic virus were seen in the small intestines in infected BLT mice [77]. Infected effector memory T cells in humanized mice are predominantly activated and proliferating [72,77], similar to findings in human AIDS patients [89,90] and SIV^+ ^non-human primates [91,92]. Brainard *et al*. similarly found an increase in activated CD4^+ ^and CD8^+ ^T cells (Ki-67^+ ^or CD27^-^) in HIV-1-infected BLT mice [42]. While central memory T cells are CCR5^- ^and are not infected by CCR5-tropic HIV-1, these cells serve as an important reservoir for replenishment of both the central memory and effector memory T cell populations [93].

Pathogenesis of HIV-1 has been implicated to be due to immune depletion in the GALT, and increased translocation of bacteria or bacterial products such as lipopolysaccharide (LPS) may lead to enhanced immune activation and AIDS. HIV-1^+ ^humans and SIV^+ ^sooty mangabeys have higher levels of LPS in the bloodstream than uninfected controls, and it was hypothesized that LPS translocated across the gut [94]. HIV-1^+ ^RAG-hu mice also exhibit increased levels of bacterial LPS in the bloodstream [51]. This was shown to be specific to HIV-1 infection, since treatment with a chemical that induces intestinal permeability did not result in higher LPS in the blood [51]. These experiments illustrate the utility of humanized mice to further investigate preliminary findings in humans and non-human primates. In addition, HIV-1^+ ^RAG-hu mice showed increased levels of activated CD8^+ ^T cells whether or not high levels of LPS were detected in the plasma [51]. Increased numbers of activated CD8^+ ^T cells in HIV-1^+ ^mice were correlated with enhanced CD4^+ ^T cell inversion [51].

Two studies have shown that CD8^+ ^T cells respond to HIV-1 infection in similar fashion as seen in humans. Sato *et al*. showed that in HIV-1-infected hNOG mice the memory CD8^+ ^population preferentially expands while the naïve population remains constant [66]. Sun *et al*. demonstrated that in HIV-1^+ ^BLT mice, the frequency of CD8^+^CXCR4^+ ^cells in the mesenteric lymph nodes and GALT decreased while the CD8^+^CCR5^+ ^population expanded. Overall, CD8^+ ^T cells expanded in the GALT and mesenteric lymph nodes and most of these cells had an effector memory phenotype (CD27^-^CD45RA^-^) [45]. Taken together, these findings indicate that HIV-1 induces a state of generalized immune activation in the gut lymphoid tissues, similar to findings in humans [89].

Human macrophages can be detected in the hNSG brain at 26 weeks post-engraftment after intrahepatic injection of human HSCs into newborns [75]. Animals were not perfused in this report, and this makes interpretation of these findings difficult, since blood cells may be detected in perivascular spaces. However, subsequent work by the same group showed limited histological data from perfused animals providing evidence for human cell engraftment in the meninges and perivascular spaces [76]. Further, intraperitoneal injection of CCR5 tropic HIV-1 leads to detection of low numbers of p24^+ ^cells in the hNSG brain (non-perfused samples) [75], possibly due to similar mechanisms of HIV-1 entry into the human brain which is thought to take place via trafficking of infected monocytes [95]. Meningitis, meningoencephalitis, and neuroinflammation were detected in a subset of animals [75]. The frequency and severity of these symptoms were increased in animals depleted of human CD8^+ ^T cells, indicating that the human immune response may be able to at least partially control neuro-invasion or neuropathogenesis in humanized mice [75]. Follow-up work by the same group has shown detection of brain abnormalities specifically in HIV-1^+ ^humanized mice as detected by live animal imaging and post-mortem immuno-staining [76]. They concluded that systemic HIV-1 infection leads to a disruption of the normal humanized mouse brain architecture [76]. As methods to improve mouse humanization continue to evolve, we expect that penetration of human immune cells to the brain will also likely increase; accompanied by higher levels of neuro-pathogenesis. Thus, humanized mice show promise for studies of the mode of viral penetration of the brain as well as pathologies associated with HIV-1 brain infection, and may additionally be useful to develop new methods to block neuro-invasion by HIV-1.

Zhang *et al*. recently showed that productive HIV-1 infection leads to activation of human plasmacytoid dendritic cells (pDCs) in the spleen and bone marrow of RAG-hu mice [63]. Activation of pDCs was followed by an increased rate of activation and apoptosis in CD4^+ ^T cell populations. Normal pDC levels were maintained despite infection of these cells, but the functionality of these cells was impaired as measured by IFN-α production and the ability to respond to TLR7 and TLR9 agonists [63].

The mechanisms by which HIV-1 induces CD4^+ ^T cell depletion and AIDS are not fully understood [93]. Garg *et al*. have recently shown that a point mutation in HIV-1 gp41 (V38E) that alters cell-to-cell fusion activity of HIV-1 envelope has no effect on the ability of the virus to replicate in BLT mice, as measured by plasma antigenemia [80]. However, CD4^+ ^T cell depletion was significantly reduced in mice infected with the V38E strain at later time points. The authors present evidence that caspase-dependent bystander apoptosis was efficient with wild-type virus, but attenuated in V38E virus.

An area of viral pathogenesis that has yet to be explored in humanized mice is the effect of secondary infections by bacterial, viral, and fungal pathogens which contribute frequently to AIDS-related mortality. Some pathogens associated with complications in AIDS patients, such as the herpesviruses EBV, KSHV and CMV as well as hepatitis B and C viruses have already been characterized for infection in humanized mice. A summary of these agents, as well as the humanized mouse models they have been studied in and appropriate references is found in Table 1.

In summary, HSC-humanized mice produce a large variety of human T cell types, and HIV-1 targets various subpopulations of these cells similarly to what is seen in humans. Further, HIV-1 is able to penetrate into the GALT and possibly into the brain, which are major sites of AIDS pathogenesis. The ability to produce large numbers of humanized mice and at a relatively inexpensive cost will allow for further expansion of our understanding of how HIV-1 penetrates these organs and how organ function is compromised by infection. While we are still in an exploratory phase of determining whether or not humanized mice truly recapitulate human AIDS pathogenesis, we look forward to the future when novel discoveries will be made in humanized mice and then confirmed in human patients.

### Human immune responses against HIV-1 in humanized mice

One of the most exciting developments of HSC-humanized mice is the capacity to develop human primary immune responses against specific agents. However, human adaptive immune responses in HSC-engrafted mice directed towards HIV-1 have been somewhat disappointing thus far; the reasons for this finding are not well understood. It appears that the ability to generate immune responses against HIV-1 may be fundamentally different than against other agents, because human immune responses against other antigens tend to be readily detectable in terms of both frequency and potency, such as after challenge with EBV [17,27-30], KSHV [32], HSV-2 [34], or Dengue virus [35,96] or after vaccination with *H. influenzae *B conjugate vaccine [46], tetanus toxin [17,97], or Hepatitis B Virus surface antigen [97]. This is generally not the case for HIV-1 (see below). Following is a summary of what has been reported in the literature in regards to human immune responses to HIV-1 in humanized mice.

Baenziger *et al*. reported a frequency of only 1 in 25 RAG-hu mice with detectable human antibodies against HIV-1 [44], Gorantla *et al*. reported 0 of 17 in RAG-hu mice [46], and our unpublished results were similar (0 of 16 in RAG-hu). Sango *et al*. reported detection of human IgG specific to HIV-1 gp120 and Gag in RAG-hu mice, but the frequency was not reported [64]. Watanabe *et al*. demonstrated human antibody responses against HIV-1 in 3 of 14 hNOG mice [43], and Sato *et al*. found the same in 2 of 7 hNOG mice [66]. The Watanabe report showed that antibodies targeted either gp120 or p24 antigens [43]. In contrast, Brainard *et al*. reported that 9 of 9 BLT mice had detectable anti-HIV-1 human antibodies after 12 weeks of infection. It is interesting to note that no responses were detected in BLT until after 5 weeks post-infection, and that 10 weeks were required for a majority to seroconvert [42]. A separate report showed detection of human IgG specific to HIV-1 proteins by western blot in 3 of 4 intrarectally-infected BLT mice [45]. A slow humoral response has also been shown against Dengue viral infection of RAG-hu mice, wherein the earliest humoral responses were detected at 2 weeks (1 of 10 animals assayed), 3 of 8 were positive by 6 weeks, and all 8 of 8 were positive by 8 weeks [35]. Baenziger *et al*. mentioned that testing was only performed on samples that were at least 3 weeks post-infection, and that the lone positive sample was from 6 weeks post-infection. Thus, it is possible that testing was performed too early to detect animals that would later become positive. However, our own unpublished ELISA testing has included at least 6 RAG-hu animals through 21 weeks (all negative) and so a slowly developing response is not likely the difference between the RAG-hu and BLT models. However, these results should be viewed in light of many variables that exist between the above studies, including the use of cord blood or fetal liver samples for transplants, the various mouse strains, the virus strains used to infect animals, the time points after engraftment at which infection took place and when samples were collected for serology, and the methodologies used to detect antibody responses. We feel that the amount of data currently available is insufficient to make definite conclusions about which models may be superior (if any) for analyzing anti-HIV-1 antibody responses.

Follow-up work with Dengue virus in RAG-hu mice has shown that ~40-60% of RAG-hu animals produce human anti-Dengue antibodies, depending upon the experiment (n > 50 infected animals). Thus, it is likely that there are fundamental differences in the antibody responses to different agents in humanized mice and that more rapid and profound T cell depletion associated with HIV-1 infection in humanized mice likely plays a role in weak antibody responses against HIV-1 as compared to more robust responses seen in humans and in humanized mice to other antigens. Interestingly, Sango *et al*. reported detection of gp120- and Gag-specific IgM and IgG in RAG-hu mice and evidence is presented that intrasplenic injection of HIV-1 results in an increased frequency of anti-HIV-1 antibody responses as compared to intravenous or intraperitoneal infection [64]. Since only the mean O.D. reading of an ELISA test is reported, the frequency of these responses is unclear, and additionally the sample sizes were small for this experiment (n = 6 for intrasplenic injection, but only n = 2 for intravenous and intraperitoneal injection as controls). However, there was a clear correlation between the number of infected splenocytes and enhanced anti-HIV-1 antibody production. Nevertheless, these data are promising that RAG-hu mice are capable of producing strong anti-HIV-1 antibody responses, and we look forward to seeing if anti-HIV-1 T cell responses will also be detectable after intrasplenic infection.

Brainard *et al*. reported in the BLT model that ELISPOT assays could detect human IFN-γ production from T cells in response to HIV-1 peptides in 4 of 6 mice, representing two different human tissue donors. No *in vivo* responses were detected earlier than 9 weeks post-infection, and gag and nef peptides were common targets, as seen in humans [98]. Intracellular cytokine staining assays further confirmed the ELISPOT results and showed that both CD4^+ ^and CD8^+ ^T cells reacted to produce IFN-γ [42]. A similar report by Gorantla *et al*. showed specific T cell responses by both CD4^+ ^and CD8^+ ^T cells to gag (but only weak, if at all, to env) in hNSG mice [71]. These reports mark the best evidence to date that HSC-engrafted mice can produce T cell responses against HIV-1, although as mentioned above these models can be used to study T cell responses against a variety of other viral pathogens. A similar study conducted by An *et al*. in RAG-hu mice failed to detect T cell responses using a similar ELISPOT assay for detection of responses against gag and nef [58]. Anti-HIV-1 T cell responses have not been reported to date in the RAG-hu model, although 4 animals were also tested by Baenziger *et al*. [44]. The mechanisms for any true differences seen in human immune responses between different humanized mouse models are not yet clear, but it has been hypothesized that the presence of human thymic tissue grafts in BLT mice may provide a more appropriate stromal environment for human T cell selection [42] and the available data support this idea for HIV-1 infections. However, detection of anti-HIV-1 T cell responses in other models that lack human thymic stromal cells (hNSG, hNOG, and RAG-hu mice) to EBV, HCV, and other immunogens [17,28,29,37,47] would argue that a stromal environment is not necessary to generate human T cell responses in general in humanized mice, suggesting a unique property of HIV-1.

HIV-1 vaccines have yet to be tested in humanized mice. A major advantage of this system is that animals can be exposed to virus by various routes and then tested for sterilizing immunity to the virus. However, since human adaptive immune responses to the virus are weak it is likely that improvements in the potency of the immune system against HIV-1 will need to be made before vaccine efficacy studies will be plausible. However, if the mechanism for weak immune responses is in fact due to rapid helper T cell loss, then it is possible that vaccines may elicit stronger responses than the live virus itself due to presentation of antigen without accompanying helper T cell loss.

A recent report by O'Connell *et al*. has shown that human T cells can be expanded in RAG-hu mice by introduction of hIL-7 via lentiviral gene transfer. T cells formed a larger percentage of human leukocytes, and similar proportions of CD4^+ ^and CD8^+ ^T cells were found in hIL-7 expressing mice as compared to control animals. Although it is possible that hIL-7 administration may lead to enhanced cellular immunity, the functionality of T cell responses were not examined in this report. Total serum IgM levels increased in hIL-7 treated animals, although the capacity to mount specific antibody responses to ovalbumin or HIV-1 did not change. Although levels of viremia were not statistically different in hIL-7 expressing mice, the total number of human T cells remained high in the spleen despite systemic HIV-1 infection [52]. These data indicate that IL-7 should be further explored as a possible mechanism to restore T cell levels in HIV-1 patients without increasing viral load. These interesting results may also lead to an improved model of humanized mice, possibly one in which anti-HIV-1 T cell responses will be more robust.

Another area yet to be examined is HIV-1 latency in humanized mice. The virus uses many mechanisms to hide in the host, but integrated provirus in a transcriptionally repressed state in resting memory T cells (reviewed in [99]) and extracellular virions tethered to the surface of follicular dendritic cells [100] contribute extensively to the latent reservoir. These models could be useful to discover new ways to induce an exit from latency as we move towards finding a cure for HIV-1.

### Anti-HIV-1 drug testing in humanized mice

Several anti-HIV-1 drugs have been tested for efficacy in HSC-engrafted mice, using both the RAG-hu and BLT models. In some reports, the effects of targeted drugs on a previously established HIV-1 infection were studied and in other reports the ability of antiviral drugs to prevent infection were analyzed. Some studies have employed FDA-approved drugs such as emtricitabine + tenofovir disoproxil fumarate (RT inhibitors) or AZT + lamivudine + indinavir (2 RT inhibitors + protease inhibitor, respectively) [77,78], or emtricitabine + tenofovir disoproxil fumarate plus an experimental strand transfer inhibitor (L-870812) [59]. The most recent study to examine the effects of drugs on a pre-existing infection has employed an experimental TAT peptide inhibitor which blocks the interaction of cellular cdk2 with the TAR element [50] and is currently being studied in humans [101].

Denton *et al*. have published two reports on pre-exposure prophylaxis in BLT mice using vaginal [77], rectal [78], or intravenous [78] exposure to HIV-1. In the vaginal challenge report, emtricitabine + tenofovir disoproxil fumarate (RT inhibitors) were administered orally 2 days prior to challenge, 3 hours prior to challenge, and 4 days post-challenge. 5 of 5 animals receiving drugs were entirely protected from infection with a CCR5 tropic strain, while 7 of 8 control animals became infected [77]. Successful HIV-1 replication in challenged mice in both of the Denton *et al*. reports was assayed by a wide variety of parameters including plasma viral load, proviral DNA amplification, plasma antigenemia, FACS staining to detect CD4^+ ^T cell loss, and in situ hybridization. Thus, this initial study has provided evidence that clinicians should administer anti-retrovirals to women who are at a high risk of HIV-1 infection via vaginal intercourse. In the second Denton *et al*. report, the ability of RT inhibitors to prevent rectal and intravenous transmission was examined [78]. In this study the same RT inhibitors were used; in this case the drugs were administered for seven consecutive days and with exposure to HIV-1 on the third day. 9 of 9 animals rectally exposed receiving drugs were entirely protected from infection with a CCR5 tropic strain, while 12 of 19 control animals became infected. This experiment is similar in nature to a recent clinical trial testing these same drugs (Truvada) as a pre-exposure regimen designed to test for protection against HIV-1 transmission in men who have sex with men or transgender women who have sex with men. That study showed that oral administration of Truvada resulted in a 44% reduction in HIV-1 transmission [102]. Although Denton *et al*. used intraperitoneal injection of Truvada versus oral administration in the clinical trial, the results are very promising that humanized mice can be useful to perform pre-clinical testing. The reasons for the differences in protection rates between the two studies are not clear, but the increased levels of human target cells in mucosal tissues in humans vs. humanized mice and the possible presence of other sexually transmitted diseases in human patients may contribute to lower protection rates in humans. Intravenous challenge with HIV-1 was performed in an identical time frame as in the rectal and vaginal challenge experiments outlined above. In this case, 7 of 8 mice administered drugs were protected from infection, while 6 of 6 control mice were infected. The lone animal to become infected did not exhibit RT mutations that would explain enhanced susceptibility to the virus. In addition, when 4 animals were infected intravenously followed by a 7-day regimen of drugs beginning 24 hours after exposure, all animals became infected but with delayed kinetics [78].

Neff *et al*. have shown that oral administration of either raltegravir (integrase inhibitor) or maraviroc (CCR5 entry inhibitor) prevents vaginal transmission of HIV-1 in RAG-hu mice. Protection rates were measured by attempts to detect viral RNA or DNA in the blood and by monitoring of CD4^+ ^T cell counts in the peripheral blood. 6 of 6 RAG-hu mice were protected from transmission with each drug, while 8 of 8 control mice were infected [62]. Taken together, these reports are very promising for effective prevention of HIV-1 transmission in humans for various routes of viral exposure.

In the other three reports, the effects of anti-HIV-1 drugs on a previously established infection were analyzed. Choudhary *et al*. showed that a combination of the RT inhibitors emtricitabine, tenofovir disoproxil fumarate, and an experimental strand transfer inhibitor (L-870812) was able to both reduce viral load as well as to rescue from CD4^+ ^T cell loss when administration was begun 10-20 days after intravenous infection of RAG-hu mice with JR-CSF (dose of virus not reported). Although limited numbers of animals were used in these experiments, it is notable that viremia was still detectable at most time points during the drug administration phase. Upon discontinuation of the drugs, viremia rebounded, and CD4^+ ^T cell levels once again dropped [59]. This finding demonstrates that viable virus was indeed present despite administration of antiviral drugs for 2-3 months. Interestingly, emergence of known resistance mutations developed in 2 of 6 mice and viral rebound occurred; this finding is discussed further in the section of viral evolution (see below).

Sango *et al*. administered a classical HAART cocktail of AZT + lamivudine + indinavir beginning 1 week after intrasplenic infection (dose range of 800 to 8000 TCID_50_) of RAG-hu mice with strain JR-CSF [64]. They found a nearly 300-fold reduction in the number of infected splenocytes which was accompanied by a 300-fold decrease in viremia. CD4^+ ^T cell loss was almost entirely prevented as well. The Choudhary *et al*. and Sango *et al*. reports clearly demonstrate that administration of anti-retroviral drugs in RAG-hu mice results in a decrease in viral load and prevention of [64] or rescue from [59] CD4+ T cell loss. However, it is important to note that both studies have utilized time points of drug administration that are considerably earlier than those used in humans, where detection of seroconversion rarely happens in the range of 7-20 days.

Van Duyne *et al*. studied the effects of cdk2 inhibitors that prevent interaction with the TAR element in RAG-hu mice [50]. Their data showed that some experimental peptide inhibitors were able to lower viremia to levels comparable to those achieved by AZT. It is interesting to note that proviral DNA copy number appeared to be substantially higher in peptide inhibitor-treated mice as compared to AZT-treated mice (about 6 × higher) and somewhat higher even than untreated HIV^+ ^mice (nearly 2 × higher). However, no statistical analysis was provided and only single time points were analyzed, so it is unclear if the difference was significant. The ramifications of possibly increasing proviral load due to inhibition of viral gene expression are not clear. This type of study shows the utility of humanized mice to identify potential complications before use in humans and to explore the mechanisms and possible side effects.

Luo *et al*. have recently shown that administration of the highly neutralizing human monoclonal antibody 2G12 is effective at reducing viremia in HIV-infected RAG-hu mice. This report also showed that an engineered 2G12 dimer was more stable and had greater viral inhibition than the monomeric form [53]. Antibodies were either administered in purified form, or via "backpack tumors" into CD34-engrafted mice. While intravenous challenge with HIV-1 in immunized mice still resulted in successful viral transmission, viral loads were suppressed. The mechanism of action of these antibodies has not yet been examined, but subtle aspects of humanized mouse engraftment are important to consider. RAG-hu mice are not expected to produce human complement since production predominantly takes place in liver cells; human liver cells are not present in RAG-hu mice. Human NK cells are also not effectively produced in humanized mice unless human IL-15 is administered [103,104]. Complement-mediated lysis of virions and complement and NK cell-mediated lysis of infected cells may thus be defective in this system when human antibodies are administered. These studies would indicate that human monoclonal antibodies may be another option for administration to HIV^+ ^patients to prevent progression to AIDS, especially since many patients fail to develop neutralizing antibodies on their own.

Taken together, these studies show that both FDA-approved and experimental treatments can be effective at blocking HIV-1 replication and helper T cell loss in humanized mice. In addition, the rationale for administration of anti-HIV-1 drugs to humans who are at high risk for HIV-1 exposure has been strengthened. Thus, humanized mice represent an important model to test unproven anti-HIV-1 drugs and proven drugs for new applications.

### Anti-HIV-1 gene therapy testing in humanized mice

Gene therapy represents a novel method to prevent and/or treat HIV-1 infections at multiple levels of the viral life cycle [104,105]. Current work in the anti-HIV-1 gene therapy field includes such strategies as blocking viral entry, blocking viral gene expression or expression of host cell factors needed for entry or replication by RNA interference [106], production of neutralizing antibodies against HIV-1, introduction of specific antiviral genes such as the APOBEC family of cytosine deaminases [107] and TRIM5α [108], and the targeting of these potential therapies to specific cell types and/or to hematopoietic stem cells [109]. Lentiviral vectors, ironically based upon the HIV-1 genome itself, have been extensively studied as gene delivery vehicles to human hematopoietic cells including HSCs [110]. These vectors are capable of genomic integration and long-term gene delivery, and do not appear to be accompanied by the leukemic events that can occur with oncoretroviruses [111]. A host of clinical trials are already underway to test various novel gene therapeutic strategies to control HIV-1 infection [112].

Gene therapy of HSCs holds high promise for a lifelong infection such as with HIV-1 because gene-modified stem cells have the potential to produce HIV-1-resistant progeny cells for long periods after initiation of therapy. Continual production of HIV-1-resistant progeny cells has the potential to provide a selective event wherein a reservoir of resistant helper T cells are maintained; thus development of AIDS may be indefinitely delayed [113]. Since HSCs are used to engraft the latest generation of humanized mice, it is only natural that several reports have emerged in which the human HSCs have been gene-modified prior to engraftment. However, there is also a certain level of concern that stem cell gene therapy poses a greater risk due to previous experience with oncoretroviral gene therapy of humans with SCID, as noted above. Thus, many efforts are also directed at modification of mature human T cells and monocytes despite the fact that these modified cells will naturally deplete during the lifetime of a patient. A summary of strategies tested thus far in the new generation of humanized mice is found in Table 3.

**Table 3:** Gene therapeutic strategies used to control HIV-1 infection in the new generation of humanized mice

Humanized mouse model	Strategy	Findings	Reference
RAG-hu mice	Lentiviral vector transduction of HSCs with anti-*nef *shRNA construct	Lentivirally-transduced HSCs can engraft in RAG-hu mice. *Ex vivo* challenge with HIV-1 showed preferential protection of transduced cells.	[60]

hNOG mice	Lentiviral vector transduction of HSCs with gene encoding neutralizing human antibody to HIV-1 (2G12)	Lentivirally-transduced HSCs can engraft in hNOG mice. Antibody was produced; viremia was ~70-fold reduced and number of infected splenocytes was reduced ~200-fold.	[68]

hNOG mice	Disrupt CCR5 gene in HSCs by zinc finger nucleases	~17% of all alleles in the HSC population were gene modified; engraftment was still successful. Gene-modified cells were positively selected during HIV-1 infection.	[74]

BLT mice	Targeted delivery of an anti-CCR5 siRNA to human lymphocytes *in vivo*	CCR5 expression was silenced and plasma viral load was decreased ~30-fold relative to controls. No CD4 T cell depletion noted through 55 days.	[81]

BLT mice	Lentiviral transduction of HSCs with anti-CCR5 shRNA construct	CCR5 expression was silenced in a variety of cell types and tissue sites. Protection against infection was measured ex vivo.	[79]

RAG-hu mice	RNA-based aptamers used to neutralize virus and/or to deliver anti-*tat*/*rev *siRNA to infected cells	Viral load decreased relative to controls; helper T cell depletion blocked. Combination therapy more effective than aptamer alone.	[54]

hNOG mice	Targeting of siRNAs to block expression of CD4, CCR5, or viral RNAs to mature human T cells	Viral load was lower after treatment, despite a moderate and transient effect on receptor knockdown. CD4 T cell levels preserved.	[69]

hNOG mice	Lentiviral vector transduction of HSCs to express antisense RNA to *env*	Only a low percentage of cells were transduced (4-11%); no effect on viral load; virus mutated targeted *env *sequences.	[70]

hNOG mice	Lentiviral transduction of mature CD4 T cells to produce viral entry inhibitor	Transduced cells expressing entry inhibitor expanded relative to non-transduced or control transduced cells, indicating protection.	[125]

In 2008, Ter Brake *et al*. used an established lentiviral vector to express an shRNA targeting the *nef *gene sequence by transducing HSCs prior to engraftment to produce RAG-hu mice [60]. While no *in vivo* challenges were performed in this study, they did show that gene-modified HSCs can still engraft in immunodeficient mice and that multi-lineage hematopoiesis still occurs. It is interesting to note that while transduction efficiencies were similar between the shRNA vector and a control vector only expressing GFP, the level of engrafted cells containing the shRNA vector was significantly lower than cells containing the control vector. This finding indicates that high level expression of shRNAs in human HSCs may interfere with engraftment and/or hematopoiesis. When RAG-hu derived cells were infected *ex vivo*, substantial protection from HIV-1 infection was noted preferentially in cells receiving the shRNA vector. Taken together with our results with NLENG1-IRES (see above) [57], these findings suggest that *nef *is a candidate target to block viral replication and cell death.

Joseph *et al*. used a lentiviral vector to deliver the gene encoding a highly neutralizing anti-HIV-1 human monoclonal antibody (2G12) into HSCs, followed by engraftment into hNOG mice [68]. They also found successful engraftment, followed by secretion of the 2G12 antibody. Upon challenge with HIV-1, viremia in gene therapy-treated hNOG mice was found to be 70-fold lower as compared to untreated animals and the number of HIV^+ ^splenocytes was reduced 200-fold. Since many HIV^+ ^patients fail to produce neutralizing antibodies this method may be useful to boost immunity in those already infected.

Recent success with possibly curing a human patient of HIV-1 infection via a bone marrow transplantation using homozygous CCR5Δ32 donor cells [114] has generated renewed interest in finding ways to interfere with CCR5 expression, especially since no known immunological defects are associated with the Δ32 allele. Various gene therapy strategies are currently being investigated as a novel method to treat HIV^+ ^patients by manipulating cells to downregulate CCR5 expression in order to possibly prevent progression to AIDS by protecting cells that would normally be susceptible to the virus. Such a strategy was employed by Holt *et al*. using zinc-finger nucleases (ZFN) to target the CCR5 gene in human HSCs, followed by transplantation into hNOG mice [74]. Transplantation efficiency was not affected by ZFN treatment, and genetically modified cells were positively selected after HIV-1 challenge, despite a relatively low level of successful gene targeting (estimated at 17% of total CCR5 alleles disrupted, with a lower level of cells having biallelic disruption). Viremia levels were lower and human T cells persisted at higher levels, indicating that the strategy was effective. Shimizu, et al reported that the BLT model can also be used to produce gene-modified cells using a lentiviral vector to transduce HSCs [79]. Their strategy was to target the cellular co-receptor CCR5 by delivery of a construct encoding a shRNA, and they showed successful knockdown of the cellular gene in T cells and monocytes/macrophages and in a variety of lymphoid tissues and also the GALT. Protection against HIV-1 challenge was again shown, but only *ex vivo*. CCR5 represents an excellent target for HIV-1 gene therapy because while viral genes can readily mutate to escape from therapy, cellular genes do not readily mutate.

Neff *et al*. recently reported on testing a novel anti-HIV-1 drug in RAG-hu mice [54]. They used RNA-based aptamers to target HIV-1 gp120, either alone or conjugated to an anti-*tat/rev *siRNA designed to block early viral gene expression in infected cells. The aptamer binds to gp120 on virions and neutralizes infectivity of particles, but can also bind to gp120 on the surface of infected cells. In conjunction with the antiviral siRNA the aptamer can be used to target delivery of the siRNA payload directly to infected cells. Their results showed that use of the aptamer alone or the aptamer-siRNA combination resulted in a drop in HIV-1 viremia by multiple logs, and that helper T cell levels were preserved. Further, the aptamer-siRNA combination provided a longer effect as compared to the aptamer alone, indicating that viral replication was inhibited.

Some studies have been performed wherein gene therapy was performed on mature human blood cells in humanized mice. Kim *et al*. targeted nanoparticles to a lymphocyte-specific marker in BLT mice; nanoparticles carried an anti-CCR5 siRNA which was accompanied by silencing of CCR5 expression for at least 10 days [81]. Although treated mice still developed a productive infection with HIV-1, the mean plasma viral load was decreased about 30-fold relative to control vector-transduced mice and no CD4^+ ^T cell loss was noted in treated mice for up to 55 days while CD4^+ ^T cell loss was detected with the control vector.

Kumar *et al*. delivered siRNAs to mature T cells in hNOG mice using a single-chain antibody to target CD7^+ ^cells [69]. siRNA targets included either CD4, CCR5, a combination of siRNAs to HIV-1 *vif *and *tat*, or a combination targeting CCR5+*vif*+*tat*. Using PBLs for transplantation, they showed that CD4 could be effectively downregulated by siRNA targeting. This resulted in lower levels of detectable HIV-1 p24 following *ex vivo* infection. Targeting of CCR5 was highly effective at reducing p24 levels in the blood in PBL-engrafted mice at day 5 post-infection, while p24 levels rose to within 1 log of the control vector by 13 days post-infection indicating that CCR5 downregulation was transient. CD4^+ ^T cell loss in treated animals was mild compared to animals receiving the control siRNA. The triple combination of CCR5+vif+tat siRNAs was highly effective at blocking virus replication for both days 5 and 13. CD4 knockdown was moderate after intravenous injection of the siRNA (~50% by FACS staining) and CCR5 knockdown was also moderate (~66% by Q-RT-PCR). *Ex vivo *infection of cells with CCR5 knockdown exhibited HIV-1 replication at about 50% levels as compared to the control vector. Viremia was highly suppressed by siRNAs against *vif *and *tat*, and CD4^+ ^T cell loss was largely prevented by the same.

### HIV-1 evolution in humanized mice

Evolution of HIV-1 *in vivo* allows the virus to escape from selective pressures including antiviral drugs and host immune responses. In addition, evolution of the *env *gene to change from the use of CCR5 as a co-receptor to use of CXCR4 is correlated with faster progression to AIDS [115,116]. The ability to recapitulate the process of viral evolution in humanized mice would allow for development of models that could potentially predict what would take place in humans. Choudhary *et al*. examined the effects of a combination of anti-HIV-1 drugs targeting reverse transcriptase (tenofovir disoproxil fumarate and emtricitabine) and the strand transfer inhibitor L-870812 in HIV-infected RAG-hu mice [59]. They found that 2 of 6 animals developed known resistance mutations in reverse transcriptase or integrase, with resistance becoming detectable as early as 1 month post-infection. Two animals died prematurely; so the incidence of drug resistance mutations may be higher.

A recent study has focused on human APOBEC proteins and their ability to induce hypermutations in the HIV-1 genome in hNOG mice [73]. Animals were infected with the molecular clones JR-CSF or *vif*-deficient JR-CSF, however vif-deficient virus was unable to replicate as assessed by undetectable RNA viral load, provirus, and p24^+ ^cells in spleen. Subsequently, sequencing analysis focused on the *pol *region were performed on samples obtained from animals infected with wild-type virus. It was found that G-to-A mutations in proviral DNA were significantly more common than other types of mutations in the HIV-1 genomes of infected humanized mice, indicating that APOBEC proteins are active *in vivo* against wild-type HIV-1. By way of contrast, G-to-A mutations were less common in plasma RNA sequences as compared to proviral sequences, indicating that hypermutated genomes were less fit for replication.

RAG-hu mice were infected with the CCR5 tropic molecular clone JR-CSF and analyzed for mutations in the *env *gene for up to 44 weeks post-infection without selective pressures from drugs or RNA silencing [65]. As mentioned above, anti-HIV-1 immune responses in RAG-hu mice have been low to undetectable and thus the selective pressure of the host immune response was likely weak in this study. Nonetheless, the mean rate of divergence of HIV-1 in RAG-hu mice was found to be similar to what was seen in human cohorts during a similar time period [117,118]. Several mutations were found in common across the group of infected mice, and included the loss of glycosylation sites and substitutions in the CD4 binding site. One animal developed *env *variants that exhibited the ability to use CXCR4 as a co-receptor, despite being infected with a molecular clone known to use CCR5 as a co-receptor. This data provide evidence that the transition from usage of CCR5 to CXCR4 is indeed as a result of viral mutation and not due to transmission of CXCR4-using viruses which can emerge later during infection [118].

The SCID-hu-PBL mouse model was used extensively to test anti-HIV-1 strategies and yielded much data [119]. A recent report using hNOG mice similarly humanized with mature human PBMCs analyzed a strategy currently under investigation in humans in which an antisense payload targeting *env *is introduced via adoptive T-cell therapy [70]. Previous reports have shown that HIV-1 can readily escape from RNA silencing [120], and this was found to be the case in humanized mice when *env *is targeted [70].

In a similar adaptation of the SCID-hu-PBL model, Mukherjee *et al*. performed an experiment to parallel a clinical trial experiment wherein an anti-HIV-1 *env *antisense RNA was introduced into PBMC-humanized hNOG mice via a lentiviral vector [70]. However, HIV-1 had already been shown to mutate in response to this antisense RNA and so the purpose of these experiments was more to examine development of these mutations in an animal system. Human T cells were transduced with the vector prior to engraftment, although only moderate levels of transduced cells were used (~4-11%) in order to mirror the clinical trial where only a low transduction efficiency is currently feasible. Animals were then challenged with one of two HIV-1 strains; NL4-3 (CXCR4 tropic) with a 100% match with the *env *target sequence and an NL4-3 derivative expressing the BaL envelope (CCR5 tropic) and hence a mismatched *env *target sequence. As expected, no differences in viral titer were detected since only a low percentage of T cells were transduced. Pyrosequencing of individual viral genomes was performed and sequences were analyzed relative to input viral sequences. Enriched A-to-G transitions were detected in the *env *target sequence region in animals receiving the antisense payload, as were viral genomic deletions in the same region. These findings were in accordance with previous work done using the same vector in a human T cell line in tissue culture [121]. G-to-A transitions also occurred at a high rate, but these changes were likely due to APOBEC-induced mutations (see below). Interestingly, for reasons which are still unclear the increase in *env *mutations was only noted in animals challenged with the CCR5-tropic envelope strain, despite the antisense RNA being imperfectly matched with the target sequence.

## Conclusions

There are many areas of HIV-1 biology that are still poorly understood, including the mechanisms by which HIV-1 causes AIDS and why some patients fail to develop AIDS. We have yet to produce a vaccine that can effectively prevent infection; nor do we have antiviral drugs that can cure an infection or that are fully refractory to viral resistance. The major roadblock to most of these unresolved problems has been the lack of suitable animal models in which to study them. The new generation of humanized mice is the most physiologically relevant system to date that allows for direct studies of HIV-1 itself as it interacts with human hemato-lymphoid cells in a complex three-dimensional environment. Humanized mice are relatively inexpensive to produce, can be worked with under conditions that are available at many institutions, and produce many aspects of HIV-induced pathogenesis directly on human cells; none of these factors apply to non-human primate research. The biggest hurdle yet to be overcome is to refine the models so that human adaptive immune responses are more robust so that vaccine efficacy experiments can be performed. Nevertheless, the data that have been generated in less than five years since the initial reports of HIV-1 infection in these new models provide much optimism that humanized mice will be useful to answer many of these unsolved questions and problems.

## List of abbreviations used

AIDS: acquired immunodeficiency syndrome; BLT: bone marrow-liver-thymus; CCR5: C-C chemokine receptor type 5; CXCR4: C-X-C chemokine receptor type 4; EBV: Epstein-Barr virus; FACS: fluorescence activated cell sorting; GALT: gut-associated lymphoid tissue; γc: common gamma chain receptor; HAART: highly active anti-retroviral therapy; HBV: hepatitis b virus; hCMV: human cytomegalovirus; HCV: hepatitis c virus; HIV-1: human immunodeficiency virus type 1; HSC: hematopoietic stem cell; HSV-2: herpes simplex virus type 2; HTLV-1: human T cell leukemia virus type 1; KSHV: Kaposi's sarcoma herpesvirus; NOD: non-obese diabetic; PBL: peripheral blood leukocyte; Prkdc: protein kinase DNA catalytic; Rag: recombinase activating gene; SCID: severe combined immunodeficiency; shRNA: short hairpin ribonucleic acid; siRNA: short interfering ribonucleic acid; TCID_50 _: tissue culture infectious dose required to infect 50%

## Competing interests

The authors declare that they have no competing interests.

## Authors' contributions

MRR is a former BS student at Brigham Young University. MRR conducted extensive literature reviews and wrote the sections on antivirals and immune responses. BKB wrote the remaining portions of the manuscript. Both authors read and approved the final manuscript.
